# Seroprevalence of SARS-CoV-2 Antibodies in Children and Adults in St. Louis, Missouri, USA

**DOI:** 10.1128/mSphere.01207-20

**Published:** 2021-02-03

**Authors:** Brittany K. Smith, Andrew B. Janowski, Jonathan E. Danis, Ian B. Harvey, Haiyan Zhao, Ya-Nan Dai, Christopher W. Farnsworth, Ann M. Gronowski, Stephen Roper, Daved H. Fremont, David Wang

**Affiliations:** a Department of Pathology & Immunology, Washington University School of Medicine, St. Louis, Missouri, USA; b Department of Pediatrics, Washington University School of Medicine, St. Louis, Missouri, USA; c Department of Biochemistry & Molecular Biophysics, Washington University School of Medicine, St. Louis, Missouri, USA; d Department of Molecular Microbiology, Washington University School of Medicine, St. Louis, Missouri, USA; University of Kentucky College of Medicine

**Keywords:** COVID-19, SARS-CoV-2, serology, seroprevalence, St. Louis

## Abstract

Reported coronavirus disease 2019 (COVID-19) case counts likely underestimate the true prevalence because mild or asymptomatic cases often go untested. Here, we use a sero-survey to estimate the seroprevalence of IgG antibodies against severe acute respiratory syndrome coronavirus 2 (SARS-CoV-2) in the St. Louis, MO, metropolitan area in a symptom-independent manner. Five hundred three adult and 555 pediatric serum/plasma samples were collected from patients presenting to Barnes-Jewish Hospital or St. Louis Children’s Hospital between 14 April 2020 and 12 May 2020. We developed protocols for in-house enzyme-linked immunosorbent assays (ELISAs) using spike and nucleoprotein and used the assays to estimate a seroprevalence rate based on our samples. Overall IgG seropositivity was estimated to be 1.71% (95% credible interval [CI], 0.04% to 3.38%) in pediatric samples and 3.11% (95% CI, 0.92% to 5.32%) in adult samples. Seropositivity was significantly lower in children under 5 years of age than in adults, but rates between adults and children aged 5 or older were similar. Of the 176 samples tested from children under 4 years of age, none were positive.

**IMPORTANCE** This study determined the percentages of both children and adult samples from the greater St. Louis metropolitan area who had antibodies to SARS-CoV-2 in late April to early May 2020. Approximately 1.7 to 3.1% of the tested individuals had antibodies, indicating that they had previously been infected by SARS-CoV-2. These results demonstrate that the extent of infection was about 10 times greater than the number of confirmed cases at that time. Furthermore, it demonstrated that by 5 years of age, children were infected to an extent similar to that of adults.

## Abstract (precis)

This study determined the percentages of both children and adult samples from the greater St. Louis metropolitan area who had antibodies to SARS-CoV-2 in late April to early May 2020.

## INTRODUCTION

Severe acute respiratory syndrome coronavirus 2 (SARS-CoV-2) was first detected in December 2019 in Wuhan, China, when several local health facilities reported patients suffering from pneumonia of an unknown cause (1). Since then, the virus has rapidly spread around the world and on 11 March 2020 was declared a pandemic by the World Health Organization. The disease caused by SARS-CoV-2, coronavirus disease 2019 (COVID-19), develops symptoms after an average incubation period of 5.2 days (2). Clinical manifestations most often include fever, cough, and fatigue but can also range from severe viral pneumonia and multiorgan dysfunction to asymptomatic infections (2). Patients who are asymptomatic or present with mild symptoms may not seek medical attention, leading to underestimation of the true prevalence (3).

Sero-surveys can estimate the cumulative incidence of SARS-CoV-2 infection in a symptom-independent manner (4), offering valuable data to inform national and local public health policies. Estimates from seroprevalence studies for locations across the United States around April and May 2020 have varied from 1 to 20% (5–10), emphasizing the need for regionally specific data. The first known COVID-19 case in Missouri was reported on 7 March 2020 and located in the St. Louis County region. By 1 May, a total of 6,332 positive cases had been reported in the St. Louis metropolitan area, equal to 0.226% of the population (https://slu-opengis.github.io/covid_daily_viz/).

The role of children in the spread of SARS-CoV-2 is unclear. Lower prevalence is generally reported in younger children than in adults. This general trend is also observed in St. Louis, with reported pediatric rates in late May of only 0.03% for children aged 0 to 9 years and 0.1% for children aged 10 to 19 years (https://stlcorona.com/). There is evidence that children may be infected at lower rates than adults (11–13) and that they are less likely to experience severe symptoms (14–17), although the relative contribution of each to lower reported rates is unclear. Asymptomatic individuals seem to account for up to 50% of SARS-CoV-2 infections, and the rate may be even higher in children (18, 19). Asymptomatic individuals, including children, show evidence of high viral shedding and likely contribute to the rapid spread of the disease (20–26). The goal of our sero-prevalence study was to determine the extent of SARS-CoV-2 infection in adult and pediatric cohorts from the St. Louis metropolitan area early in the outbreak.

## RESULTS

### Sensitivity and specificity of ELISA.

**FIG 1:**
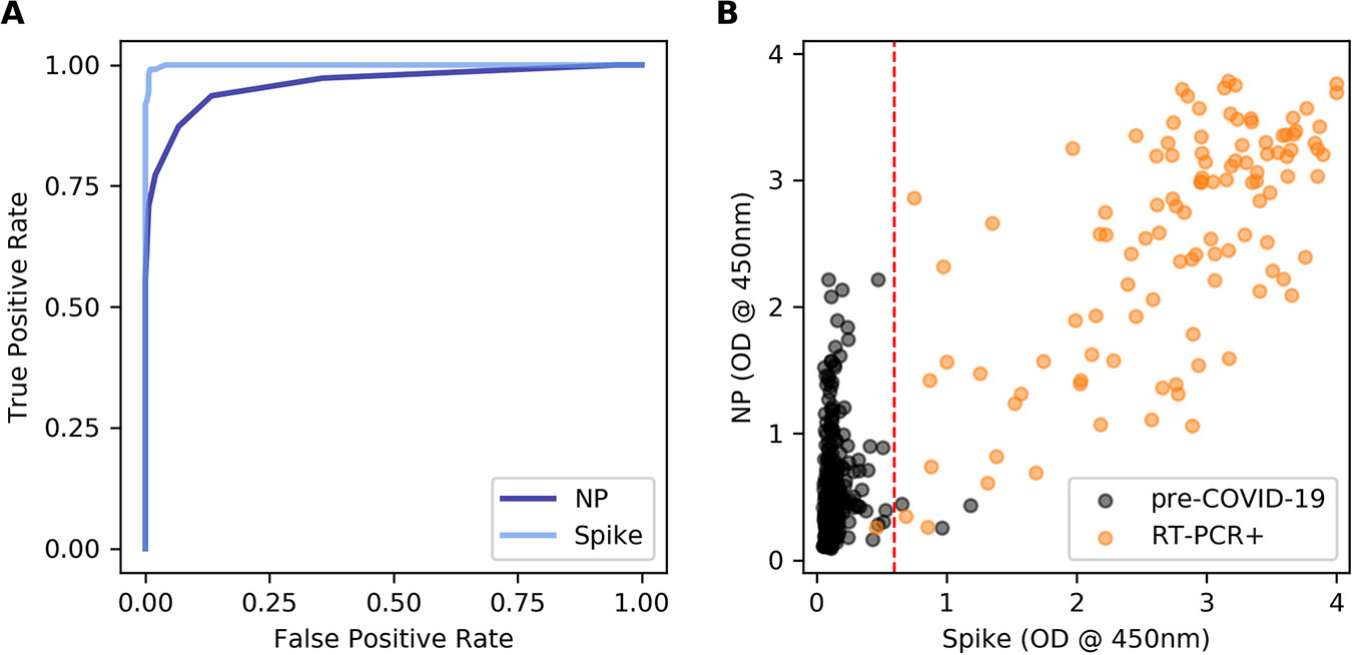
Optimization of spike and NP ELISA protocols. (A) ROC curves comparing the true-positive and false-negative rates of spike and NP IgG ELISAs for different OD cutoffs. (B) Comparison of NP and spike ELISA results for 300 negative (pre-COVID-19) samples and 110 RT-PCR-positive samples. The dashed line shows the spike ELISA cutoff used for seroprevalence calculations (pre-COVID-19 sera mean plus 4 SD).

We performed receiver operating characteristic (ROC) analysis for the spike and nucleoprotein (NP) antigens to determine diagnostic thresholds for IgG enzyme-linked immunosorbent assays (ELISAs) (Fig. 1). Negative pre-COVID-19 samples had greater cross-reactivity to NP (mean, 0.516; standard deviation [SD], 0.557) than to spike (mean, 0.142; SD, 0.114) (Fig. 1B). ELISA sensitivity and specificity were estimated with a Markov chain Monte Carlo (MCMC) scheme. Area under the curve (AUC) values were 0.999 and 0.961 for the spike and NP assays, respectively. The optimal cutoff point for each assay was selected as the number of SD above the pre-COVID-19 sample mean that maximized the Youden index (sensitivity plus specificity minus 1).

Based on this criterion, the optimal cutoff for the spike IgG ELISA was equal to the average optical density (OD) value of prepandemic samples plus 4 SD, resulting in 98.2% sensitivity (95% CI, 97.4% to 99.9%) and 98.7% specificity (95% CI, 95.7% to 100%). The NP IgG ELISA was less accurate, with 86.5% (95% CI, 80.1% to 92.8%) sensitivity and 93.1% (95% CI, 90.3% to 95.8%) specificity for the assay’s optimal cutoff of the average pre-COVID-19 OD plus 2 SD. Requiring a positive response to both antigens decreased assay performance (87.4% sensitivity and 99.7% specificity) compared to that of the spike ELISA alone, so samples with spike IgG ELISA responses greater than the spike cutoff are considered seropositive, regardless of reactivity to NP.

### Demographic and clinical characteristics.

Deidentified adult samples included 503 individuals (296 females, 207 males) with a median age of 61 years (range, 18 years to 93 years) (Table 1). The pediatric cohort included 555 different individuals (286 females, 269 males) with a median age of 9 years (range, 2 days to 17 years).

**TABLE 1:** Adult and pediatric cohort demographics

Cohort	Patient characteristic^*a*^	No. of samples tested	No. (%) of seropositive samples^*b*^
Pediatric		555	15 (2.70)
	<1	89	0 (0.00)
	1–4	118	1 (0.85)
	5–9	79	4 (5.06)
	10–14	143	6 (4.20)
	15–17	126	4 (3.17)
	Male	269	4 (1.49)
	Female	286	11 (3.85)

Adult		503	21 (4.17)
	18–30	29	1 (3.45)
	30–39	41	1 (2.44)
	40–49	57	2 (3.51)
	50–59	111	9 (8.11)
	60–69	147	7 (4.76)
	>69	118	1 (0.85)
	Male	207	9 (4.35)
	Female	296	12 (4.05)

a Numbers are patient ages in years.

b Data are number of seropositive samples and percent of total tested samples with positive response for each group.

### SARS-CoV-2 seroprevalence in St. Louis.

**FIG 2:**
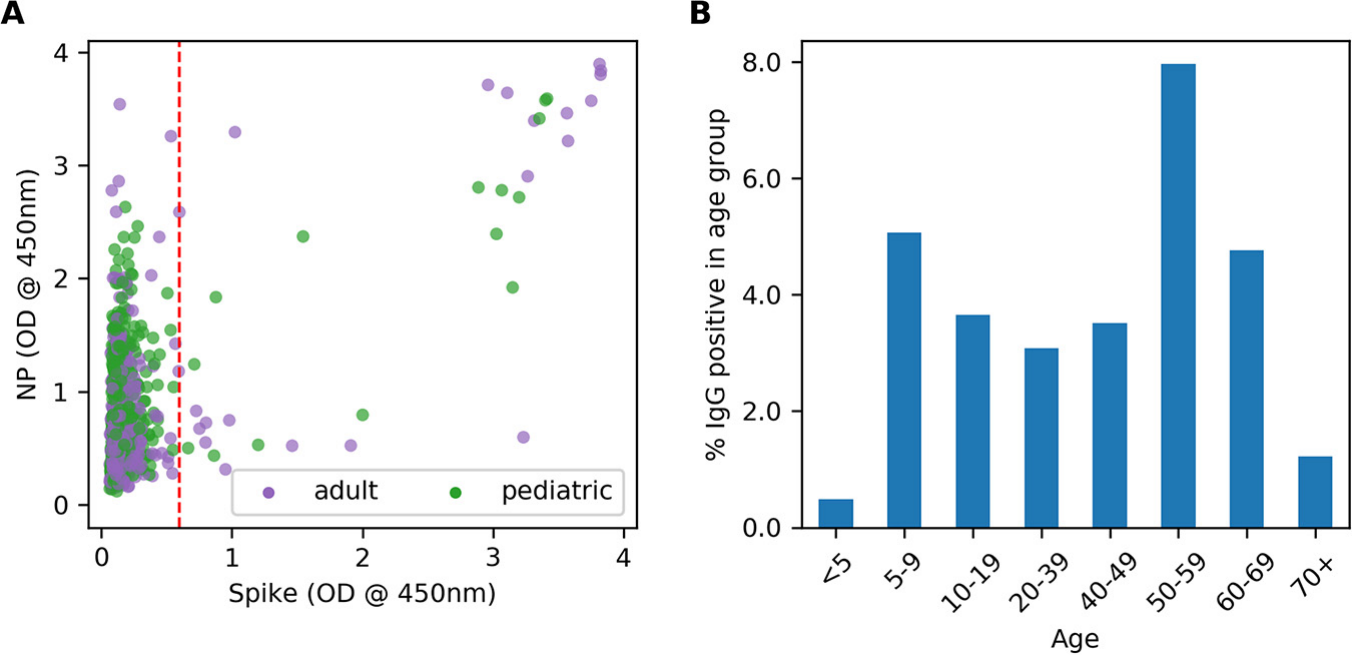
ELISA results for 503 adult and 555 pediatric samples. (A) NP and spike IgG ELISA results for 1,055 serum/plasma samples collected in April and May 2020. The dashed line shows the spike OD cutoff used to define seropositivity. (B) Frequency of seropositive samples by age group (in years).

**FIG 3:**
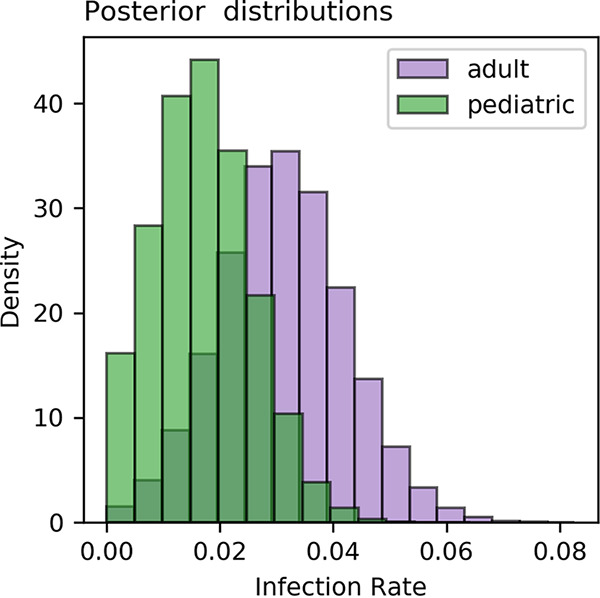
Seropositivity rate estimates for pediatric and adult cohorts. Histograms of posterior simulations of SARS-CoV-2 seroprevalence.

Spike and NP ELISA results for the 1,055 tested samples are shown in Fig. 2A. Thirty-six samples were found positive for anti-spike IgG antibodies with OD values above the cutoff value. Twenty-one of the positive samples were identified from the adult cohort (*n* = 503) and 15 from the pediatric cohort (*n* = 555). Accounting for test performance in a Bayesian regression model led to estimated seroprevalence rates of 3.11% (95% CI, 0.92% to 5.32%) and 1.71% (95% CI, 0.04% to 3.38%) for adults and children, respectively (Fig. 3). A breakdown of positive samples by demographic information is provided in Table 1 and Fig. 2B. Of the 176 samples tested in children under 4 years of age, none were positive. The youngest seropositive subject was a 4-year-old female child and the oldest a 70-year-old male.

As the St. Louis Department of Health reported an incidence rate of only 0.03% for children aged 0 to 9 years in late May, despite an overall St. Louis population incidence rate of 0.226%, we tested the hypothesis that our data provide evidence for higher seropositivity in adults than in young children. Our data suggest an estimated seroprevalence rate of 0.67% for children under 5 years, which is significantly lower than the estimated adult seropositive rate of 3.11% (*P* = 0.0032). However, the rate for children between 5 and 18 years is 3.37%, similar to the rate of adults (*P* = 0.473). Our results do not provide evidence for seroprevalence rate differences based on sex (*P* = 0.256).

## DISCUSSION

We used a serological assay to identify SARS-CoV-2 antibodies in children and adults early in the St. Louis outbreak. We observed seroprevalence rates of 1.71% among children and 3.11% among our adult cohort. A prior study estimated the seroprevalence of SARS-CoV-2 in Missouri at 2.7% using 1,882 serum samples, most from adults, collected broadly across the state in late April (27). The adult incidence rate that we report in samples from the St. Louis metropolitan area was slightly higher, consistent with a higher reported infection rate by reverse transcription-PCR (RT-PCR) in St. Louis than in the rest of Missouri (https://slu-opengis.github.io/covid_daily_viz/; https://stlcorona.com/).

We observed lower estimated seropositivity in children under 5 years than in adults, and none of the samples from children under 4 years of age were identified as seropositive. These results are consistent with a growing body of evidence that young children may be infected by SARS-CoV-2 at a lower rate than adults (28–30). One potential caveat is that due to differences in inclusion criteria of the pediatric and adult cohorts, there may be biases that limit comparative interpretation of the observed seropositivity rates. Additional studies performed with samples collected later in the pandemic are needed to better understand pediatric infection rates and inform public health protocols related to schooling.

In this study, we established and validated a robust ELISA protocol for the detection of SARS-CoV-2 antibody responses in serum/plasma samples. We found that a seropositivity criterion based on the spike IgG ELISA alone is more accurate than one based on both the spike and NP antigens, possibly due to high NP cross-reactivity to seasonal coronaviruses (31). A Bayesian model is then used to jointly estimate the specificity and sensitivity of the test along with the seroprevalence rate. In this way, our reported posterior distributions for seroprevalence account for test specificity and sensitivity while propagating uncertainty in these parameters into final seroprevalence estimates.

Our study used sera from patients obtaining medical care early in the pandemic, so it is not necessarily generalizable to the general St. Louis population. Notably, the St. Louis stay-at-home order overlapped with our sample collection period, when routine medical visits and bloodwork for healthy patients may have been limited. Individuals with severe medical conditions therefore may be overrepresented in our cohort relative to the general population.

An improved understanding of general seroprevalence in both adult and pediatric populations is critical to implementing public safety guidelines. Our results indicate that both children and adults are susceptible to SARS-CoV-2 infection and develop antibody responses. Serological studies such as this provide essential information on the risk for transmission and the immunological state of the population.

## MATERIALS AND METHODS

### Patient cohorts.

We screened 503 adult and 555 pediatric serum/plasma samples. The adult samples were residual samples sent to Barnes-Jewish Hospital for physician-ordered vitamin D testing between 27 April 2020 and 12 May 2020. Residual pediatric specimens were collected from unique outpatients presenting to St. Louis Children’s Hospital between 14 April 2020 and 8 May 2020. The most common indications for pediatric blood draw were an evaluation of allergies, a general health assessment in the emergency department (basic and comprehensive metabolic profiles), and screening/follow-up for a variety of conditions (includes orders for bilirubin, vitamin D, lipids, iron/ferritin, TSH/free T4, and HgbA1c). Specimens with orders for immunosuppressant therapeutic drug monitoring were excluded.

Assay sensitivity was evaluated with two sets of sera (*n* = 110) collected from COVID-19 RT-PCR-positive individuals that exhibited neutralization titers of >1:40 in a focus reduction neutralization assay (I. Harvey and D. Fremont, unpublished data). The first set included 35 serum samples from 16 patients being treated at the Mayo Clinic, collected 6 to 20 days after symptom onset. The remaining 75 COVID-19-positive samples, from 75 individuals following convalescence (≥2 weeks without symptoms and PCR negativity), were collected by Washington University researchers.

Assay specificity was assessed using a subset of pre-COVID-19 serum samples collected between 2007 and 2008 for prior serological studies (32, 33) and stored at –80°C. Three hundred pre-COVID-19 samples were obtained from adult (*n* = 150) and pediatric (*n* = 150) patients presenting to Barnes-Jewish Hospital or St. Louis Children’s Hospital.

This study was approved by the Human Research Protection Office at Washington University in St. Louis (approval no. 202004199 and 202004153).

### Cell line, virus, and recombinant protein.

Purified RNA from the 2019-novel CoV(nCoV)/USA-WA1/2020 SARS-CoV-2 strain was reverse transcribed into cDNA and used as the template for recombinant gene cloning. Full-length SARS-CoV-2 NP was cloned into pET21a with a hexahistidine tag and recombinantly expressed using Escherichia coli BL21(DE3)-RIL in terrific broth (bioWorld). Following overnight induction with isopropyl β-d-1-thiogalactopyranoside (GoldBio) at 25°C, cells were lysed in 20 mM Tris-HCl, pH 8.5, 1 M NaCl, 5 mM β-mercaptoethanol, and 5 mM imidazole for nickel affinity purification. Following elution in the prior buffer supplemented with 500 mM imidazole, the protein was purified to homogeneity using size exclusion chromatography, followed by cation exchange chromatography.

The SARS-CoV-2 spike ectodomain (residues 14 to 1211; GenBank accession no. MN908947.3) and influenza hemagglutinin (HA) ectodomain (subtype B/Colorado/06/2017) were cloned into pFM1.2 with an N-terminal μ-phosphatase signal peptide. The C terminus of SARS-CoV-2 spike was engineered with an HRV3C protease cleavage site (GSTLEVLFQGP) linked by a foldon trimerization motif (YIPEAPRDGQAYVRKDGEWVLLSTFL) and an 8×His Tag. The S1/S2 furin cleavage site was mutated, and 2 stabilizing proline mutations were introduced. The influenza HA construct contained a T4 fibritin trimerization domain at the C terminus (34). The plasmids were transiently transfected into Expi293F cells and purified by cobalt-charged resin chromatography (G-Biosciences) as previously described (35, 36).

### ELISA.

We developed two ELISAs based on trimeric spike and NP proteins. Our protocol was previously optimized based on serial dilution results of 16 negative controls and 122 positive controls as described in the work of Harvey et al. (unpublished data). Briefly, 96-well MaxiSorp plates were coated with 2 μg/ml of purified antigens in 50 mM Na_2_CO_3_ (70 μl) overnight at 4°C. Plates were then washed with phosphate-buffered saline (PBS)–0.05% Tween 20 and blocked with 200 μl 1× PBS–0.05% Tween 20–1% bovine serum albumin (BSA)–0.02% sodium azide for 2 h at room temperature. Serum/plasma samples were diluted 1/500 in blocking buffer. Diluted samples were then added to plates (50 μl/well) and incubated for 1 h at room temperature. Bound IgG was detected using horseradish peroxidase (HRP)-conjugated goat anti-human IgG (at 1/5,000). Following 1 h of incubation, washed plates were developed with 50 μl of a 1-Step Ultra TMB ELISA solution and quenched with 2 M sulfuric acid, and the absorbance was read at 450 nm. The results of independent technical replicates (*n* = 3) were averaged for each sample. The ELISA response of each sample to the influenza HA ectodomain was also evaluated as a control.

### Cutoff value and statistical analysis.

Sensitivity and specificity values were calculated for spike and NP ELISAs from the positive- and negative-control sera. Cutoff values were determined by ROC analysis focused on maximizing both the sensitivity and the specificity of the assay. To assess plate-to-plate variation, the same 2 negative controls (pre-COVID-19 sera) and 2 positive controls (sera from SARS-CoV-2 PCR-positive individuals) were included on every ELISA plate. The coefficient of variation was less than 20% in all cases. All blank wells had absorbance values of <0.15.

Parameters were estimated with a Bayesian framework using a Markov chain Monte Carlo (MCMC) method implemented in python using the PyStan package (37). Five independent chains of 10,000 iterations each were simulated, with the first 1,000 iterations corresponding to the burn-in. Noninformative beta(1) priors, along with a binomial likelihood function, were used for sensitivity, specificity, and prevalence. Convergence was assessed by visual inspection of time series plots and Gelman and Rubin $\hat{R}$ statistics (38). Posterior distributions for each parameter were described using means and 95% posterior credible intervals.

Bayesian hypothesis testing was done to examine the effects of age and sex on SARS-CoV-2 seroprevalence. The *P* values that we report are Bayesian *P* values (39). We calculate the χ^2^ discrepancy measure between generated data sets and the expected values drawn from the posterior distribution. The Bayesian *P* value is the probability that the χ^2^ discrepancy measure based on the replicated data were more extreme than the observed data. Differences were considered to be statistically significant at a *P* of <0.05.
